# Microscopic Monitoring of Erythrocytes Deformation under Different Shear Stresses Using Computerized Cone and Plate Flow Chamber: Analytical Study of Normal Erythrocytes and Iron Deficiency Anemia

**DOI:** 10.1155/2018/6067583

**Published:** 2018-10-24

**Authors:** Mohamed A. Elblbesy

**Affiliations:** ^1^Department of Medical Biophysics, Medical Research Institute, Alexandria University, Egypt; ^2^Department of Medical Laboratory Technology, Faculty of Applied Medical Science, University of Tabuk, Saudi Arabia

## Abstract

Erythrocytes deformation is one of the exciting properties of erythrocytes. It is still under investigation by many of the researchers in different disciplines. The clinicians and researchers are still looking for a simple and efficient method to monitor and tracking the erythrocytes deformation. This research article represented a microscopic technique that could be a helpful tool in evaluation and studying of erythrocytes deformation under different shear stresses. This technique was used to compare the deformation of normal erythrocytes and iron deficiency anemia. Elongation index was calculated and used in the quantitative analysis of erythrocytes deformation. It was found that the deformability of normal erythrocytes was higher than that of iron deficiency anemia. Normal erythrocytes and iron deficiency anemia showed strong negative correlations with the mean cell volume and the mean cell hemoglobin concentration under different shear stresses. This study introduces more understanding of the erythrocytes deformation by using a simple microscopic technique. The elongation index could be used as a measurable parameter to evaluate the deformability of the erythrocyte in normal and abnormal cases.

## 1. Introduction

Erythrocytes deliver oxygen to all body tissue and allow carbon dioxide to move from tissues to the lung. They are biconcave disks. This shape confers unique mechanical properties to erythrocytes [1–3]. Erythrocyte deformability is the changing in its shape in response to the external force. It alters the efficiency of the transportation of blood gases [4]. The decreasing in it leads to increase in blood flow resistance and blood viscosity. It is essential to blood flow through the circulatory system [4, 5]. The deformability of erythrocytes depends on many factors such as cell geometry, viscoelasticity of cell membrane, and cell contents [6]. The surface area to volume ratio (S/V) and the hemoglobin concentration are examples of such factors that could alter the erythrocytes deformation [7, 8].

Many pathophysiological conditions affect the deformability of erythrocytes. The disorders associated with the formation of erythrocytes membrane structures and hemoglobin protein can altered erythrocytes deformability. Thalassemia and iron deficiency anemia are examples of such disorders. Thalassemia results into loss of erythrocyte deformability due to the formation of abnormal hemoglobin molecules. Also, Heinz bodies formation is associated with abnormal hemoglobin molecules, and it causes the local rigidification of the erythrocyte membrane [9–12]. Iron deficiency is a systemic disorder. It is one of the most frequent diseases throughout the world which affects a variety of different cell types. Many rheological studies suggest that erythrocyte deformability is impaired; others, however, do not confirm these observations [13–16].

Many techniques are used to study erythrocyte deformation [4]. Some of these techniques are used to evaluate the deformation of the individual cells such as micropipette aspiration and atomic force microscope [17, 18]. Others are used to study the deformation of multiple cells such as filtration method and microfluidic filtration [19–21]. These techniques usually provide deformation index only without any information about erythrocytes deformation distribution [22, 23]. The erythrocytes deformation distribution promotes the existence of cell-populations with anomalous mechanical properties [24]. Recently, developing of rheoscope enables monitoring erythrocytes deformation and its distribution and orientation [25]. Also, it provides a helpful tool to study tank-treading motion, the effect of shear stress amplitude on elongation, and the effect of shear rate on the rotational speed of tank-treading motion [22, 26].

The present work introduces a simple microscopic technique using in monitoring erythrocyte deformation under different shear stresses. It will be used in the analysis and evaluation of the deformability of the iron deficiency anemia erythrocyte (IDA).

## 2. Materials and Methods

### 2.1. Sample Collection

Twenty blood samples were collected from volunteers with IDA. Twenty blood samples were collected from healthy volunteers and were used as a control. All volunteers were of the males and aged between 35 to 45 years. All blood samples were collected on ethylenediaminetetraacetic acid (EDTA) as an anticoagulant. For each sample complete blood count (CBC) had been done. Blood indices such as mean cell volume (MCV) and mean corpuscle hemoglobin concentration (MCHC) were noted from CBC. Erythrocytes were separated by centrifugation at 3000 rpm and suspended in autologous plasma at 1 % hematocrit to be used in quantitative analysis of erythrocytes deformation.

### 2.2. Cone and Plate Flow System (CPFS)

Cone and plate flow system (CPFS) was designed and built with an inner diameter of 5 cm. It is constructed from a cone glass with a 0.5° angle from its center. The cone rotated about its central axis. It was fixed on ball bearings and placed upper to a flat plate of glass. The ball bearing was fixed on upper lid connected to the stepper motor with a rubber belt. The bottom glass plate was fixed on the bottom lid. The upper lid and bottom lid were connected by setscrews from two sides. These setscrews were used to adjust the distance between the cone and plate by moving the upper lid up and down. The rotation was controlled to produce a stable flow. This construction gave a uniform shear rate. All cells between the cone and plate were exposed to the same shear stress. All procedure was performed at a temperature of 25°C. The following equation was used to calculate the shear stress inside the CPFS:

(1) $$\begin{array}{c} \tau_{\omega }=\frac{\omega \mu }{\theta } \end{array}$$

where *τ*_*ω*_ is the shear stress, *ω* is the angular velocity of the cone, *μ* is the fluid viscosity, and *θ* is the angle of the cone.

### 2.3. Image Acquisition System and Deformation Measurement

The imaging capture system consisted of an eyepiece camera and inverted microscope. The CPFS was fixed on the inverted microscope stage. The eyepiece camera was mounted on the microscope eyepiece column enabling the transformation of images directly to the computer. The objective lens of 40X was used for erythrocytes observation. The total magnification of the image after capturing by the camera was approximately 500 X. Two dimensions images were captured by this system at a frame rate of 30 frames per second. High-resolution images (1280 × 1024 pixels) were produced, providing the opportunity for an in-depth analysis of the erythrocytes under steady shear conditions. Erythrocytes were suspended in plasma at a concentration of 1 % and incubated at 37°C for one hour before the experiment. The CPFS was filled with erythrocytes suspension gradually. The flow was initiated at shear stress in the range of 5-40 dynes/cm^2^. All procedure was performed at a temperature of 25°C. Erythrocytes images under different shear stress for control and IDA were collected at ten different locations on the CPFS. Images were transferred to the computer through USB connection. Image analysis was performed using ImageJ free software. Offline digital analysis of erythrocytes images under different shear stresses was done. The images were transferred to 8-bit images and threshold in black and white color. Ellipse contour was fitted to each cell in the image. The major (*L*_*maj*_) and minor (*L*_*min*_) axes of the cell were measured by the particle analysis tool. Deformation of erythrocytes was examined by calculating the elongation index. The elongation index (EI) was calculated as follows:

(2) $$\begin{array}{c} EI=\frac{L_{maj}}{L_{min}} \end{array}$$

where *L*_*maj*_ is the major axis length of the erythrocytes and *L*_*min*_ is the minor axis length of the erythrocyte.

### 2.4. Statistical Analysis

All data were expressed as the mean values ± standard deviation (SD). Correlation between EI and blood parameters for control and IDA was done using Pearson r correlation. Comparison of the results was made using 2-way ANOVA. Significance was taken at *p* < 0.05. The results were be analyzed using IBM SPSS Statistics for Windows, version 21.0.

## 3. Results and Discussion

**Figure 1:**
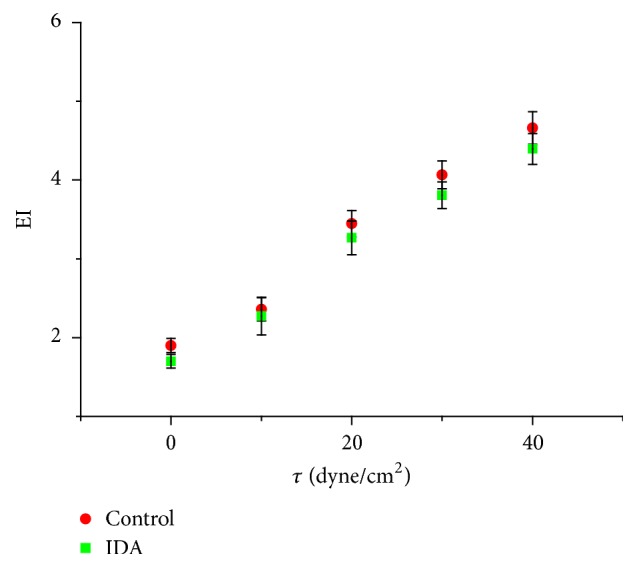
Elongation index of erythrocytes versus applied shear stress for control and IDA.

Erythrocytes change their shape according to the flow conditions [27]. It was reported that at low shear stresses erythrocytes behave as a solid particle. At sufficiently high shear stresses erythrocytes act like liquid drops change their shape into ellipsoids [28, 29]. In the present study scatter plot of EI versus *τ* reflected the linear direct relationship between them as shown in Figure 1. The differences between EI at various shear stresses was significant (*p* < 0.05) for both control and IDA when compared with 0 dyne/cm^2^. EI of control was higher than IE of IDA. The rising in EI was monitored clearly at high shear stress and the difference between EI of control and IDA became obvious. EI of control and IDA showed that under steady state of flow erythrocytes changed to an ellipsoid shape. The erythrocytes deformed gradually as shear stress increased and oriented in the direction of the flow. This could be explained as the erythrocytes cell membrane resisted the deformation and the transformation from biconcave to ellipsoid was controlled by the elasticity of the cell membrane. Comparison study between the deformation of normal and IDA was done by Vayá A et al. using ektacytometric techniques. They indicated lower EI for IDA in comparison with normal erythrocytes [30]. As monitoring in this study, the shape transformation of erythrocytes in control was more rapidly than IDA. Normal erythrocytes were more deformable than IDA. The difference between EI of IDA and control was not significant for shear stress range 10- 30 dyne/cm^2^ (*p* > 0.05). But it was significant at higher shear stress (*p* < 0.05).

**Figure 2:**
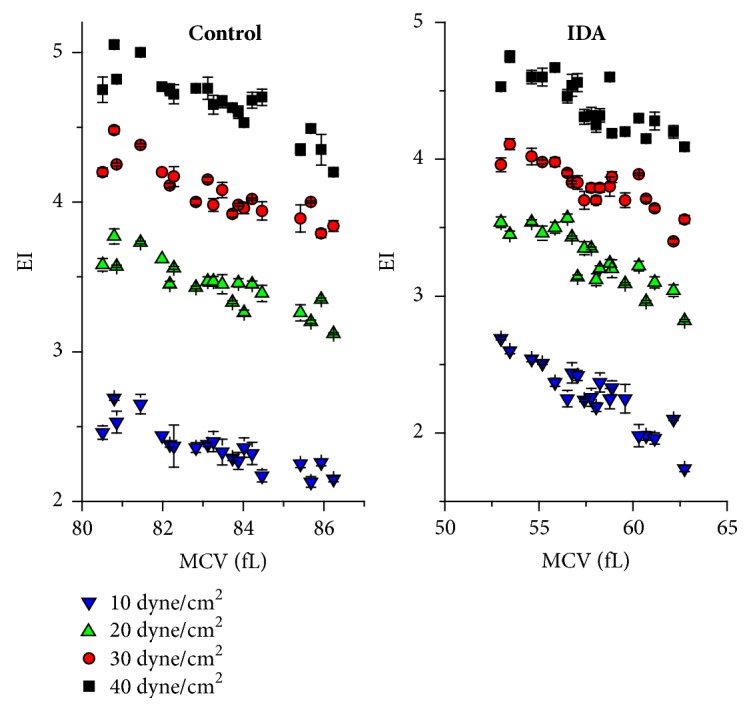
Mean cell volume negatively correlated to elongation index for control and IDA.

EI was inversely proportional to MCV. This was observed under low and high shear stresses and for control and IDA as shown in Figure 2. Under normal conditions, the erythrocytes are a biconcave shape with 8 *µ*m diameter, ~135 *µ*m^2^ surface area, and ~90 fL volume. The surface area to volume ratio (S/V) is approximately 1.5 which could be altered by osmotic pressure. These characteristics facilitate large deformation and ability of shape transformation. The biconcave disk of the erythrocyte is changed to an ellipsoid by the shearing flow [31]. Decreasing of the S/V ratio leads to reduced erythrocytes deformability. The reduction of erythrocytes deformability associates with the pathogenesis of several erythrocytes disorders including hereditary spherocytosis, hemolytic anemia, and malaria-infected erythrocytes [7, 32]. Reduction in cell volume of IDA may be the significant intrinsic factor that reduced the deformability of erythrocytes. The scatter plot Figure 2 of the IE versus MCV for IDA and control supported this assumption. Strong negative correlations were obtained for the relationships between EI and MCV under different shear stresses in control and IDA as given in Table 1.

**Figure 3:**
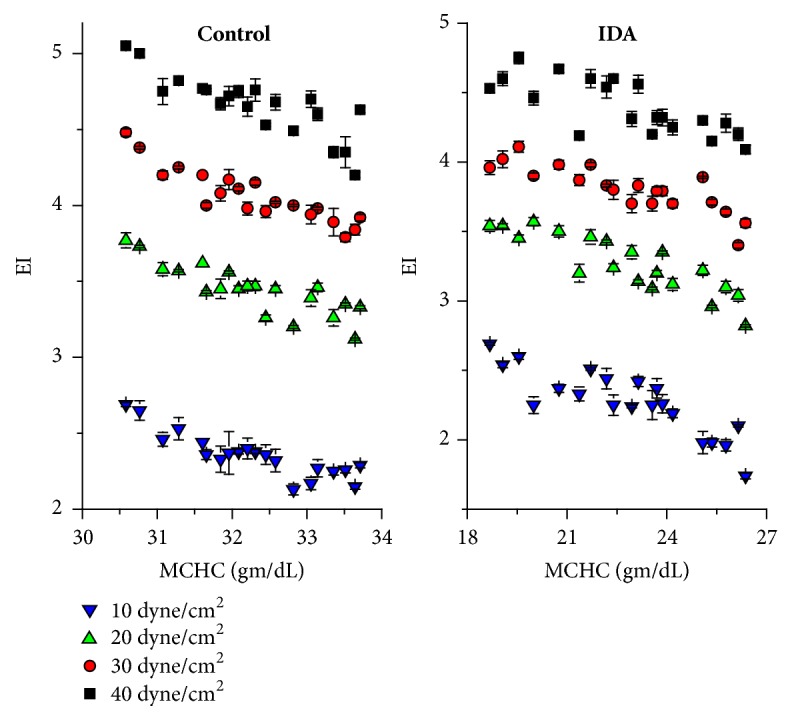
Mean cell hemoglobin concentration negatively correlated to elongation index for control and IDA.

Cytoplasmic viscosity is one of the significant intrinsic factors affecting erythrocytes deformability. This was explained as the MCHC increased in such cases [33]. Losing water from erythrocytes is another similar situation leading to decreasing of erythrocytes deformability due to a local increase in cytoplasmic viscosity [8]. In this study, MCHC correlated to EI as shown in Figure 3. Strong negative correlations between EI and MCHC were obtained for control and IDA for the whole range of shear stress under investigation.

## 4. Conclusion

This study showed efficiency and effectiveness in monitoring erythrocyte deformation using the microscopic technique. The results obtained demonstrate that this technique can be used to trace erythrocyte deformability under different physical conditions. It can be concluded that this technique offers an easy and simple way for researchers and doctors to study erythrocyte deformability.

## Figures and Tables

**Table 1:** The correlation coefficients of EI under different shear stresses with the blood indices.

EI	Control		IDA	
	MCV	MCHC	MCV	MCHC
10 dyne/cm^2^	-0.87084	-0.87689	-0.92129	-0.85997
20 dyne/cm^2^	-0.87678	-0.84504	-0.87894	-0.87274
30 dyne/cm^2^	-0.87135	-0.90976	-0.85151	-0.8425
40 dyne/cm^2^	-0.87845	-0.84543	-0.82442	-0.75954

## Data Availability

The author confirms that the data supporting the findings of this study are available within the article.
